# Hypoxia enhances indoleamine 2,3-dioxygenase production in dendritic cells

**DOI:** 10.18632/oncotarget.24098

**Published:** 2018-01-09

**Authors:** Xiang Song, Yan Zhang, Li Zhang, Wengang Song, Lixin Shi

**Affiliations:** ^1^ Department of Endocrinology, The Second Affiliated Hospital of Soochow University, Suzhou 215004, China; ^2^ Comprehensive Ward, Affiliated Hospital of Guizhou Medical University, Guiyang 550004, China; ^3^ Department of Endocrinology, Affiliated Hospital of Guizhou Medical University, Guiyang 550004, China; ^4^ Institute of Immunology, Taishan Medical University, Tai’an 271000, China

**Keywords:** dendritic cells, hypoxia, indoleamine 2,3-dioxygenase, adenosine, inflammation

## Abstract

Hypoxia-associated metabolic reprogramming modulates the biological functions of many immune and non-immune cells, and affects immune response types and intensities. Adenosine and indoleamine 2,3-dioxygenase (IDO) are known immunosuppressors, and adenosine is a hypoxia-associated product. We investigated the impact of hypoxia on IDO production in dendritic cells (DCs). We found that hypoxia (1% O_2_) enhances IDO production in DCs, and this increase was dependent on the adenosine A3 receptor (A3R), but not A2aR or A2bR. A3R blockade during hypoxia inhibited IDO production in DCs, while A2bR blockade further enhanced IDO production. Activating A2aR had no effect on IDO production. Hypoxia (1% O_2_) upregulated CD86, CD274, HLA-DR, and CD54, and downregulated CD40 and CD83 in DCs as compared to normoxia (21% O_2_). IDO inhibition in hypoxia-conditioned DCs reversed MHC-II, CD86, CD54, and CD274 upregulation, but further downregulated CD40 and CD83. Our findings offer guidance for pharmacological administration of adenosine receptor agonists or antagonists with the goal of achieving immune tolerance or controlling insulin resistance and other metabolic disorders via IDO modulation.

## INTRODUCTION

Dendritic cells (DCs) are versatile cells that initiate adaptive immune responses, maintain steady state immunity, and mediate inflammatory responses as innate immune cells [1]. DC function is generally determined by localized environmental factors. DCs are mainly tolerant under steady-state conditions, and promote the generation of immunosuppressive T-regulatory cells (Tregs) that inhibit effector CD4^+^ and CD8^+^ T cell proliferation and activation [2]. Under inflammatory conditions, DCs are stimulated to activate and mature, and can induce either innate or adaptive responses. Several self-regulatory mechanisms limit inflammatory response expansion, and are critical for immunohomeostasis maintenance.

Hypoxia is an important microenvironment factor commonly observed in many pathological contexts, including solid tumors and inflammation, as well as some normal physiological processes [3, 4]. Tissue-resident DCs and blood DC precursors, including monocytes, can be recruited to sites of inflammation or solid tumor tissues that may be hypoxic. The effects of hypoxia on DCs are unclear, possibly due to differences in DC lineages, O_2_ tensions, and time courses across experiments. DCs also exhibit distinct behaviors in the hypoxic sites of solid tumors versus inflamed joints in rheumatoid arthritis (RA) cases. DCs in solid tumors are tolerant and inhibit adaptive Th1 cells [5], whereas they stimulate inflammatory Th17 cells in the joints of RA patients [6]. Thus, understanding how hypoxia finely regulates DC function is important for the development of effective anti-hypoxia-associated disease therapies.

Tolerogenic DCs execute their immunosuppressive functions via inhibitory molecules, such as programmed death ligand receptors (PD-L1 and PD-L2), CD103, arginase, or indoelamine 2,3-dioxygenase (IDO) [7–9]. IDO, a rate-limiting enzyme in tryptophan degradation, impacts peripheral tolerance and immune regulation [10]. IDO activation may deplete local concentrations of the essential amino acid tryptophan, and tryptophan metabolites, such as kynurenine, 3-hydroxyanthranilic acid, and 3-hydroxykynurenine, can act as immunosuppressants [11]. Under normal physiological conditions, IDO production is also critical for maintenance of immunohomeostasis, fetal and oral tolerance, and an immunosuppressive environment in the gut. However, IDO overproduction is detrimental to mounting an effective immune response against tumors and some invading pathogens [12]. Some DC subsets produce more IDO than others, such as CD103^+^ DCs in the gut that express higher IDO levels than CD103^−^ DCs. Still, most DC subsets can be induced to produce IDO by immunosuppressive cytokines, such as TGF-β, immunosuppressive signals, such as CTLA-4, STAT3, and Foxo3, and some inflammatory stimuli, such as IFN-γ, IFN-α, and TLR agonists [13–17]. The role of hypoxia in regulating IDO production by DCs is as yet unclear.

Several mechanisms have been suggested to explain attenuation of hypoxia-mediated tissue inflammation and injury. Increased production of extracellular adenosine, a well-known anti-inflammatory factor, is one such self-protective response to hypoxia. Extracellular adenosine is produced from successive phosphohydrolysis of ATP by ectonucleotidases such as CD39 and CD73, which are upregulated by hypoxia [18]. The effects of extracellular adenosine are mediated by the four P1 purinergic receptors, A1, A2A, A2B, and A3. Adenosine A2a and A2b receptors mediate adenosine inhibition of inflammatory cytokine release (IL-12, IL-6, and TNF) and Th1 cell activation by DCs [19, 20]. Adenosine receptors are likely involved in hypoxia-regulated IDO production in DCs.

In some inflammatory microenvironments, such as inflamed joints in RA, hypoxia, GM-CSF and/or IL-4 may coexist, stimulating monocyte differentiation into DCs. This study explored the effects of hypoxia on IDO production in monocyte-derived DCs, and showed that hypoxia increased IDO production via adenosine A3R.

## RESULTS

### Hypoxia (1% O_2_) enhances IDO production

IDO depletes tryptophan, an amino acid essential for cell growth. IDO expression reportedly mediates T cell tolerance by DCs. Many immune microenvironment factors induce IDO production in DCs, such as IFN-γ, TNF-α, and PGE2 [21]. Given the roles of hypoxia in immune tolerance and inflammation, we evaluated IDO production in DCs during DC differentiation from monocytes under hypoxic conditions.

**Figure 1:**
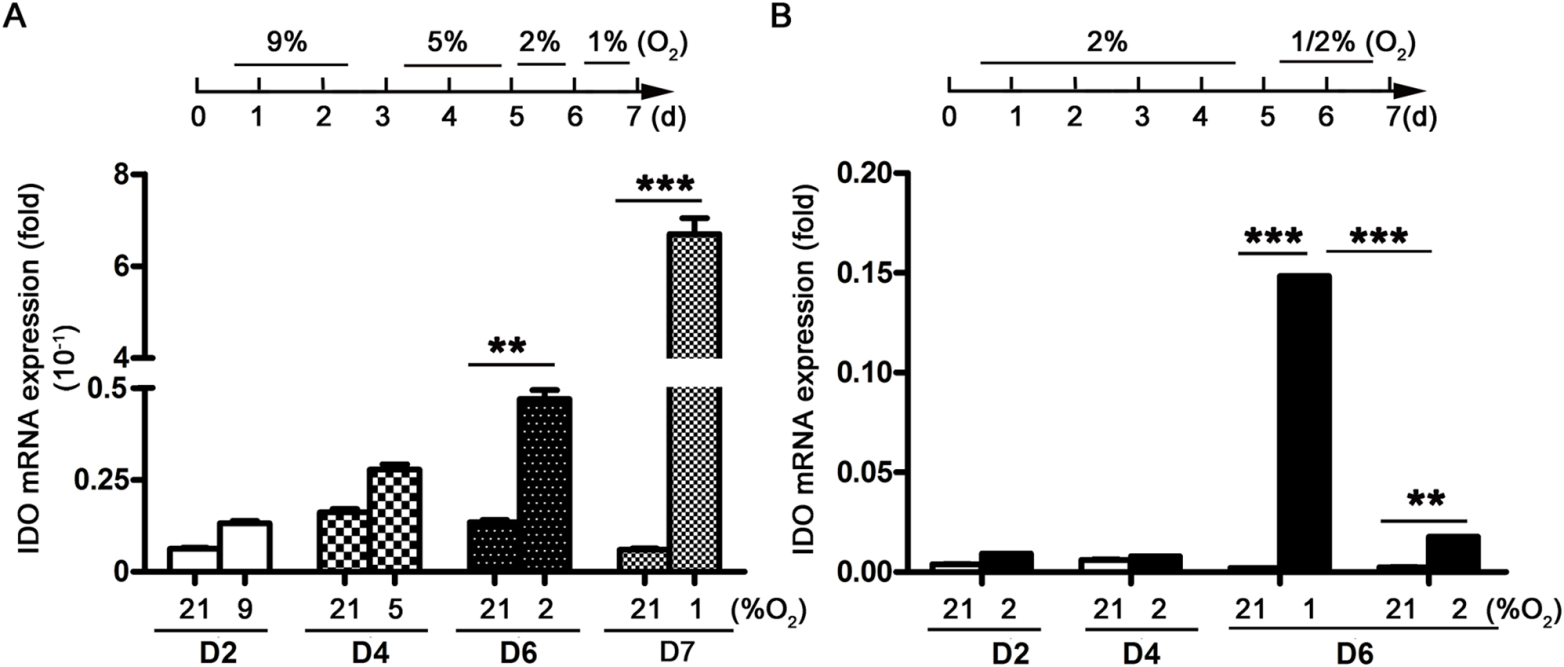
IDO production during DC differentiation from monocytes under hypoxic conditions Monocytes were cultured under conditions of normoxia (21% O_2_; controls), 9-5-2-1% O_2_ hypoxia **(A)**, or 2-1% O_2_ hypoxia **(B)** IDO expression relative to that of GAPDH was detected using real-time PCR. Data are shown as means ± SD of three replicates. ^**^P<0.01, ^***^P<0.001.

DC differentiation from monocytes via GM-CSF and IL-4 takes approximately seven days under normoxic conditions (21% O_2_). We therefore cultured monocytes in hypoxic conditions for seven days. We also cultured monocytes in sequential hypoxia conditions over seven days to mimic hypoxia in circulation (9–5% O_2_) and tissues (2–1% O2), conditions to which monocytes might be exposed during their migration from blood into tissues. Hypoxia dose-dependently upregulated IDO expression in DCs during differentiation from monocytes. The lowest O_2_ concentration tested (1% O_2_) had the strongest stimulatory effect (Figure 1A). To rule out the possibility that increased IDO production was associated with the differentiation process and not hypoxia, monocytes were cultured with 2% O_2_ for five days, followed by 1% or 2% O_2_ for the last two days. IDO expression was still stimulated by 1% O_2_, but not by 2% O_2_ (Figure 1B).

### Effects of IDO on mature DCs under hypoxic conditions

**Figure 2:**
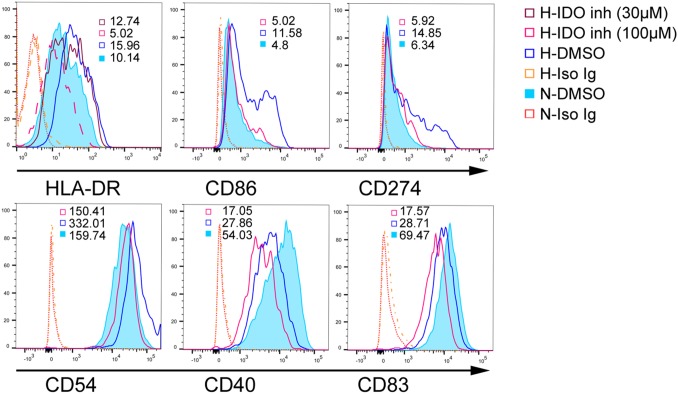
IDO effects on mature DCs under hypoxic conditions Monocytes were cultured under conditions of normoxia (21% O_2_; controls) or 2% O_2_ hypoxia for 5 d. The IDO inhibitor, 1-MT (100 or 30 μM), was added to hypoxia-conditioned DCs for another 2 d at 1% O_2_, and CD86, CD40, CD54, HLA-DR, CD83, and CD274 expression was detected using FACS. Hypoxia-conditioned DCs with isotype Ig staining were pooled from those cells treated with DMSO or 1-MT. Results are shown as relative median fluorescence intensities (normoxia- or hypoxia-conditioned DCs with specific Ig staining *vs*. normoxia- or hypoxia-conditioned DCs with isotype Ig staining) and represent three experiments.

The costimulatory and MHC phenotypes are critical for DC-mediated T cell responses. Although IDO contributes to T cell tolerance by DCs, the effects of IDO on DC maturation are still unclear. We blocked IDO production in DCs before exposure to hypoxia (1% O_2_) using an IDO inhibitor (1-MT) on day five. We detected mature DC phenotypes via flow cytometry two days later. Hypoxia (1% O_2_) upregulated MHC-II, CD86, and CD54 in DCs and inhibited CD40 and CD83 compared to normoxia (Figure 2). Hypoxia (1% O_2_) also upregulated CD274 (PD-L1), which reduces T cell proliferation and induces T cell apoptosis. Blocking IDO production in hypoxia-conditioned DCs reversed MHC-II, CD86, CD54, and CD274 upregulation, but further downregulated CD40 and CD83 (Figure 2).

### HIF-1 is non-essential for IDO production in DCs under hypoxic conditions

**Figure 3:**
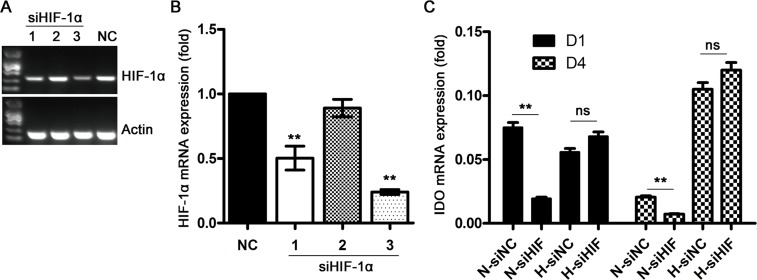
Effects of HIF-1 signaling on IDO production in DCs during hypoxia Monocytes were transfected with control (NC) or HIF-1α-specific siRNAs under normoxic conditions for 48 h, and *HIF-1α* expression was detected using real-time PCR **(A** & **B)** Knockdown efficiency was calculated by normalizing HIF-1α expression to that of Actin; **(C)** DCs were transfected with NC or HIF-1α siRNA (pooled HIF-1α siRNA_1+2_ as indicated in (B) on d 1 or d 4 for 48 h under normoxic (21% O_2_) or hypoxic conditions (5 d of 2% O_2_ then 1 d of 1% O_2_), and then IDO expression was detected relative to that of GAPDH using real-time PCR Data are shown as means ± SD of three replicates. ^**^P<0.01.

To explore the underlying mechanisms of hypoxia-induced IDO production in DCs, we evaluated the effects of HIF-1 signaling, a well-described hypoxia-induced pathway that controls expression of >100 hypoxia-associated genes. We found that HIF-1α knockdown via siRNA (Figure 3A, 3B) inhibited IDO production in DCs under normoxic conditions, but had no effect on IDO under hypoxic conditions as compared to control siRNA-treated DCs (Figure 3C). This indicated that HIF-1 signaling regulates IDO production in DCs under normoxic conditions, but is not essential for increased IDO expression during hypoxia (1% O_2_).

### Adenosine A3 receptor controls IDO production during hypoxia

**Figure 4:**
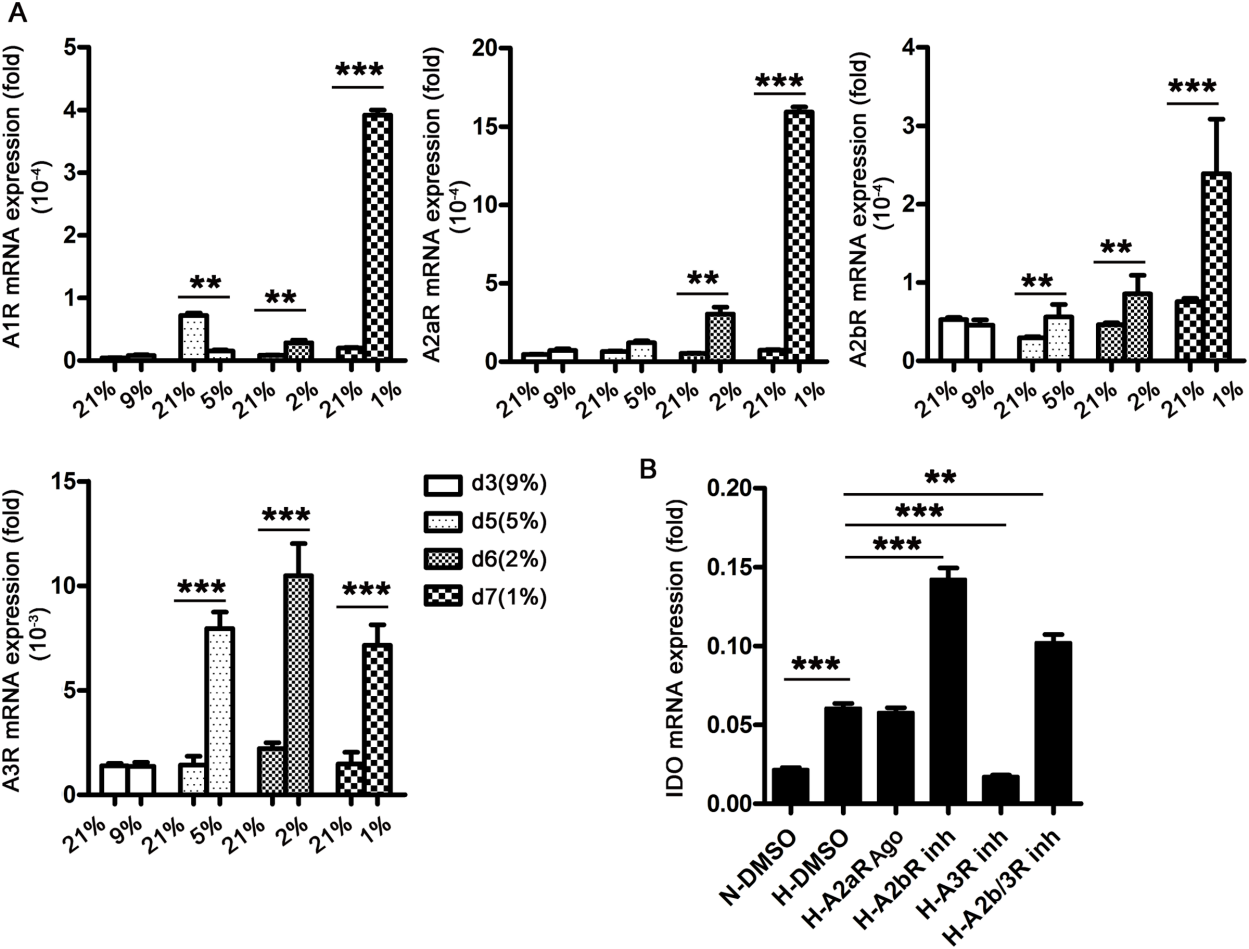
Adenosine A3R is involved in IDO production in DCs during hypoxia Monocytes were cultured under conditions of normoxia (21% O_2_; control) or 9-5-2-1% O_2_ sequential hypoxia for 7 d, and A1R, A2aR, A2bR, and A3R expression relative to that of GAPDH was detected using real-time PCR. **(A)** DCs on d 5 were treated with specific AR inhibitors or an agonist, and IDO expression relative to that of GAPDH was detected using real-time PCR. **(B)** Data are shown as means ± SD of three replicates and represent three experiments. ^**^P<0.01, ^***^P<0.001.

Extracellular adenosine is increased by tissue hypoxia. To investigate the roles of adenosine in IDO production, we dynamically detected adenosine receptor (AR) expression in DCs during differentiation. We found that hypoxia upregulated four adenosine receptors, A1R, A2aR, A2bR, and A3R. A2aR and A2bR expression increased the most at 1% O_2_ late in the differentiation process, and A3R was the most abundant at this late stage (Figure 4A). We then evaluated the roles of A2aR, A2bR, and A3R in IDO production during hypoxia (1% O_2_) using specific inhibitors of A2bR and A3R, and an A2aR agonist. A3R blockade during hypoxia inhibited IDO production in DCs, while A2bR blockade further enhanced IDO production. Activating A2aR had no effect on IDO production (Figure 4B). These results indicated that adenosine released during hypoxia (1% O_2_) promotes IDO production via A3R.

### A3R signaling modulates DC phenotype

**Figure 5:**
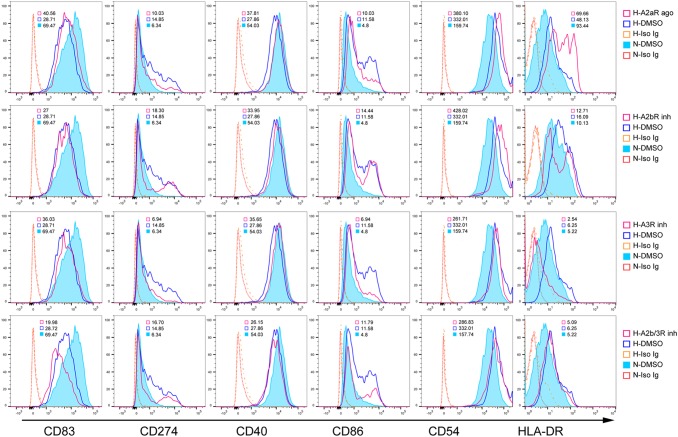
Adenosine A3R modulates DC phenotypes during hypoxia Monocytes were cultured under conditions of normoxia (21% O_2_; control) or 2% hypoxia for 5 d. A2bR or A3R inhibitors, or an A2aR agonist was added to hypoxia-conditioned DCs for another 2 d at 1% O_2_, and CD86, CD40, CD54, HLA-DR, CD83, and CD274 expression was detected using FACS. Data are shown as relative median fluorescence intensities to isotype Ig staining, and represent three experiments.

Adenosine-treated DCs are reportedly impaired in stimulating T cell responses. As costimulatory and MHC-II molecules are critical for antigen presentation by DCs, we investigated whether and which adenosine receptors were involved in IDO-upregulated CD86, CD274, MHC-II and CD54 expression. We treated DCs on day five (before 1% O_2_ hypoxia) with A2bR or A3R inhibitors, or an A2aR agonist. Similar to the effects of IDO inhibition in DCs, CD86, MHC-II, CD54, and CD274 upregulation was reversed by A3R inhibition, but was enhanced or unaffected by A2bR blockade or A2aR activation (Figure 5). Moreover, blocking A3R had no effect on CD40 and CD83 downregulation. These findings indirectly confirmed that hypoxia induced IDO production in DCs via A3R.

## DISCUSSION

Hypoxia-associated metabolic reprogramming helps determine bioenergetic needs during the immune response and other physiological and pathological processes. Hypoxia and increased extracellular adenosine are both features of inflammation and solid tumors [22–24]. We found that hypoxia divergently regulates mature DCs via extracellular adenosine-induced IDO production in an autocrine manner. The auto-regulatory effects of IDO in DCs might be associated with tryptophan metabolites, such as kynurenine, 3-hydroxyanthranilic acid, and 3-hydroxykynurenine. Our observations of crosstalk between hypoxia, adenosine receptors, and DC-produced IDO suggest a novel mechanism through which hypoxia-related metabolites in the microenvironment modulate immune responses in cooperation with other immune cells, particularly T cells. In addition to directly inhibiting effector T cell responses, IDO upregulates PD-L1 in DCs. The PD-L1/PD-1 interaction in T cells transmits an inhibitory signal that suppresses T cell proliferation and may induce apoptosis [25, 26].

Our findings also indicated that IDO upregulates CD86 and HLA-DR, the costimulatory and MHC-II molecules critical for DC-triggered effector CD4^+^ and CD8^+^ T cell responses. However, such upregulation may have no effect during extreme hypoxia, because our previous work showed that DCs did not efficiently stimulate T cell responses under <5% O_2_ conditions. Impaired T cell responses during hypoxia may be due in part to direct inhibition of IDO released from DCs or other cell types, and/or regulatory T cell activity. In contrast to effector T cells, CD4^+^CD25^+^ regulatory T cell potency is reportedly enhanced under hypoxic conditions [27, 28]. Additionally, IDO-producing DCs are essential for induction of regulatory T-cell responses [29]. Thus, in hypoxic microenvironments, such as in some solid tumors, DCs are tolerant not only due to inhibitory signals from adenosine/A2aR and A2bR, but also from autocrine IDO. This promotes regulatory T cell survival and function, but inhibits the generation of effector CD4^+^ and CD8^+^ T helper cells, which may promote tumor cell growth and expansion.

Hypoxia may also induce IDO production in non-immune cells via adenosine/A3R signaling, and this metabolic reprogramming mechanism may participate in many other pathophysiological processes. Obesity, dyslipidemia, hypertension, and type 2 diabetes are described as metabolic syndromes, which may involve inducible IDO production in adipose tissues. During chronic inflammation, a common factor in metabolic syndromes, the local hypoxic microenvironment may upregulate extracellular adenosine production. Adenosine controls insulin signals in adipose tissue, muscle, and liver [30, 31], and all four adenosine receptors are reportedly involved in glucose homeostasis, adipogenesis, and insulin resistance [32]. Increased levels of IDO-generated tryptophan metabolites, the kynurenines, may promote metabolic syndrome development via pro-oxidative, neurotoxic, and apoptotic activities, along with upregulation of inducible nitric oxide synthase, phospholipase A2, arachidonic acid, prostaglandin, 5-lipoxygenase, and leukotriene cascade [33]. Whether or not hypoxic factors increase IDO production via adenosine in metabolic syndromes is as yet unknown.

IDO can be produced by multiple immune cell types, such as macrophages, DCs, and eosinophils, as well as epithelial, endothelial, and tumor cells [34]. Regulation of IDO expression is complex. Antigen-presenting cells, such as DCs and macrophages, are preferentially stimulated to express IDO when challenged with proinflammatory stimuli, including IFN-γ, TNFα, IL-32, and PGE2 [35]. IFN-γ is an important product of activated NK and effector CD4^+^ and CD8^+^ T cells, which are induced during viral infections. TNF-α and IL-32 are also critical mediators in some inflammatory diseases, including RA, chronic obstructive pulmonary disease, and graft-vs.-host disease [36]. Along with TNFα and IL-32, hypoxic factors also promote IDO production. Thus, hypoxia-induced IDO in combination with other inflammatory stimuli may limit inflammation expansion and injury in RA as part of key immunoregulatory feedback loops.

Several signaling pathways can induce IDO production. Janus kinase 1 (JAK1) and Stat1 signaling mediate IFN-γ-induced *IDO* transcription [37]. Stat1 directly binds IFN-γ-activated sites within the IDO promoter or indirectly affects *IDO* transcription by inducing IFN regulatory factor-1 (IRF-1), which binds the *IDO* promoter at two IFN-stimulated response element sites [38]. Additionally, the JAK1/2/STAT3, protein kinase Cδ, and NF-κB pathways can also induce *IDO* expression [39, 40]. Still, the mechanisms by which hypoxia/adenosine/A3R triggers IDO expression in DCs must be further clarified. Our study suggests that decreased intracellular cAMP levels may be involved A3R-mediated IDO upregulation. Prostanoid PGD2 inhibits IDO expression via the PGD2 receptor, DP1, which increases intracellular cAMP levels that is found to be inhibitory in IDO production. This PGD2 inhibitory effect acts through DP1/cAMP/PKA/CREB signaling [41]. Extracellular adenosine relays signals by activating any of its four receptors (A1R, A2aR, A2bR, and A3R). A1R or A3R activation attenuates intracellular cAMP levels, whereas A2aR or A2bR activation elevates cAMP levels [42]. We observed that blocking A2bR further enhanced IDO expression, implying that A3R-induced IDO production is associated with cAMP/PKA pathway inhibition.

In summary, we demonstrated that hypoxia induces IDO production in human DCs via adenosine A3R, overcoming the inhibitory effects of A2bR. The relationship between hypoxia, adenosine, and IDO should be further explored in other cell types. Our findings offer guidance for pharmacological administration of adenosine receptor agonists or antagonists with the goal of achieving immune tolerance or controlling insulin resistance and other metabolic disorders via IDO modulation.

## MATERIALS AND METHODS

### Monocyte-derived DC induction and culture

CD14^+^ human monocytes from buffy coats of healthy volunteers (Department of Transfusion, Shanghai Changhai Hospital) were purified using anti-CD14 microbeads (Miltenyi Biotec, Bergisch-Gladbach, Germany) and cultured in RPMI 1640 medium (Gibco) containing 10% FBS (Gibco), 50 ng/mL GM-CSF (R&D Systems), and 10 ng/mL IL-4 (R&D Systems) under normoxic (21% O_2_, controls) or hypoxic (range from 9% to 5% to 2% to 1%, or from 2% to 1%, or 2% O_2_) conditions for seven days. In some experiments, cells were transfected with 20 nM siRNA (GenePharm) using INTERFERin (Polyplus-transfection) or treated with a specific inhibitor of IDO (1-methyl-[D]-tryptophan, 1-MT, 100 μM), an adenosine receptor inhibitor (A2bR, MRS1754 from Calbiochem; A3R MRS1191 from Sigma-Aldrich; both 10 μM) or an A2aR agonist (CGS21680, 10 μM, Calbiochem). After 48 h, cells were collected for analysis. Sequences of siRNAs of HIF-1α are: 5’-CCAGUUAUGAUUGUGAAGUUATT-3’ (sense), 5’-UAACUUCACAAUCAUAACUGGTT-3’ (antisense); 5’-GGUGUUGCGUAAAUCAUAUTT-3’ (sense), and 5’-AUAUGAUUUACGCAACACCTT-3’ (antisense); and 5’-GGAGCUAACAGGACAUAGUTT-3 (sense), and 5’-ACUAUGUCCUGUUAGCUCCTT-3’ (antisense). Procedures were approved by the ethics committee of Guizhou Medical University and informed consent was obtained from all donors in this study.

### IDO and adenosine receptor quantitative PCR analyses

Quantitative reverse transcription-polymerase chain reaction (qRT-PCR) was carried out as described previously [43]. Total RNA was extracted using TRIzol (Invitrogen). Quantitative PCR was performed on a LightCycler (Roche, Basel) using the SYBR RT-PCR kit (Takaro). Expression data from each sample were normalized to β-actin or GAPDH. Primer pairs were as follows: β-actin-FW, 5’-GGCGGCACCACCATGTACCCT-3’; β-actin-RV, 5’-AGGGGCCGGACTCGTCATACT-3’; GAPDH-FW, 5’-TGTGGGCATCAATGGATTTGG-3’; GAPDH-RV, 5’-ACACCATGTATTCCGGGTCAAT-3’; IDO-FW, 5’-AGTCCGTGAGTTTGTCCTTTCAA-3’; IDO-RV, 5’-TTTCACACAGGCGTCATAAGCT-3’; A1R-FW, 5’-TCTGGGCGGTGAAGGTGAAC-3’; A1R-RV, 5’-AGTTGCCGTGCGTGAGGAAG-3’; A2aR-FW, 5’-AGGCAGCAAGAACCTTTCAA-3’; A2aR-RV, 5’-CTAAGGAGCTCCACGTCTGG-3’; A2bR-FW, 5’-CCCTTTGCCATCACCATCAG-3’; A2bR-RV, 5’-CCTGACCATTCCCACTCTTGA-3’; A3R-FW, 5’-AGACCACCACCTTCTATTT-3’; A3R-RV, 5’-GACCCTCTTGTATCTGACG-3’.

### FACS analysis of DC phenotypes

Human DC phenotypes were assessed via flow cytometry (Becton Dickinson, LSRFortessa). Cells were incubated with fluorochrome-conjugated monoclonal antibodies (CD40-PE-Cy7, CD86-BV510, HLA-DR-APC, CD83-APC-Cy7, CD274-APC, and CD54-PE; BD PharMingen) for 30 min on ice, washed, and analyzed using FlowJo software as previously described [44].

### Statistical analyses

Statistical analyses were performed using Graphpad Prism5 software. Differences were assessed using unpaired two-tailed Student *t* tests for two groups or ANOVAs for more than two groups. Statistical significance was set at *P*<0.05.
